# Maternal exercise training attenuates endotoxin-induced sepsis in mice offspring

**DOI:** 10.1016/j.bbrep.2018.06.001

**Published:** 2018-06-14

**Authors:** Mami Yamada, Chihiro Hokazono, Mitsuharu Okutsu

**Affiliations:** Graduate School of Natural Sciences, Nagoya City University, 1 Yamanohata, Mizuho-cho, Mizuho-ku, Nagoya, Aichi 467-8501, Japan

**Keywords:** LPS-induced sepsis, Inflammatory response, Maternal exercise, Offspring

## Abstract

Regular exercise during pregnancy can prevent offspring from several diseases, such as cardiovascular diseases, obesity, and type II diabetes during adulthood. However, little information is available about whether maternal exercises during pregnancy protect the offspring from infectious diseases, such as sepsis and multiple organ dysfunction syndrome (MODS). This study aimed to investigate whether maternal exercise training protects the offspring from endotoxin-induced septic shock in mice. Female C57BL/6 mice performed voluntary wheel exercises during pregnancy. All dams and offspring were fed normal chow with sedentary activity during lactation and after weaning. At 10-week-old, mice were intraperitoneally injected a lethal (30 mg/kg) or nonlethal (15 mg/kg) dose of lipopolysaccharide (LPS), following which the survival of mice that were administered a lethal dose was monitored for 60 h. Plasma, lung, and liver samples were collected 18 h after the injection to evaluate the cytokine concentration or mRNA expression from those administered a nonlethal dose. Although maternal exercise training could not prevent lethality during an LPS-induced septic shock, it significantly inhibited the LPS-induced loss of body weight in female offspring. Regular maternal exercise significantly inhibited the mRNA expression of the LPS-induced inflammatory cytokines, such as interleukin-1β (IL-1β) and interferon-γ (IFN-γ), in the plasma and liver. Thus, maternal exercise inhibited the LPS-induced inflammatory response in female offspring, suggesting that regular exercise during pregnancy could be a potential candidate of the onset of sepsis and MODS in offspring.

## Highlights

• Maternal exercise inhibited the LPS-induced loss of body weight in female offspring.
• Regular exercise during pregnancy inhibited the LPS-induced inflammatory cytokines.
• Maternal exercise could be a potential candidate of the onset of sepsis in offspring

## 1 Introduction

Maternal behavior during pregnancy affects the embryonic environment, which in turn, affects the prenatal development of offspring and leads to their predisposition to various chronic diseases in adulthood, such as cardiovascular diseases, hypertension, obesity, and type II diabetes [1], [2]. This nongenetic impact has been obtained from the developmental programming hypothesis, which proposes that fetal and early neonatal environmental stimuli acting during the critical windows of development, such as fetal and/or early postnatal periods, can permanently alter the cell/tissue structure and function [3].

Regular exercise is a potent stimulus to enhance mammalian health. In addition, recent research has highlighted that regular exercise during pregnancy contributes to offspring health in adulthood. Maternal exercise during pregnancy has been reported to improve the metabolism via an increase in the lean mass and a decrease in the fat mass percentage in male offspring [4]. Another recent study has reported that maternal exercise during pregnancy decreases the endothelium-independent vascular function in adult swine offspring [5]. Furthermore, we have recently reported that regular exercise during pregnancy prevents the maternal high-fat diet–induced hypermethylation of the peroxisome proliferator-activated receptor-γ coactivator-1α (pgc-1α) gene and the age-dependent metabolic dysfunction in offspring [6]. These findings suggest that regular exercise during pregnancy is a determining stimulus for the predisposition of offspring to prevent cardiovascular and metabolic diseases. However, whether maternal exercise affects the offspring inflammatory response in adulthood remains unknown.

Inflammation can be categorized into two types: acute and chronic. Acute inflammation is the initial response to harmful stimuli that are attained by the activation of immunological cells and the induction of inflammatory cytokines. Notably, a dysregulated inflammatory response leads to persistent tissue damage, various pathophysiological disorders, or death [7]. Sepsis is characterized by the dysregulation of an inflammatory response primarily following a bacterial infection [8]. In the US, the incidence of sepsis is reported to be > 1.5 million cases per year [9]. The sepsis-induced mortality rate is estimated to be 30%, which increases with age from 10% in children to 40% in the elderly [10]. However, the exact reason for an uncontrolled inflammation and death in some patients with sepsis remains unclear.

Lipopolysaccharide (LPS), which induces acute inflammation and sepsis-like conditions, accelerates the release of various humoral mediators, particularly inflammatory cytokines that play major roles in the induction of systemic inflammation and the development of sepsis [8]. Among the inflammatory cytokines, tumor necrosis factor-α (TNF-α), interferon-γ (IFN-γ), and interleukin-1β (IL-1β) are detected in the blood of patients with sepsis and induce septic shock-like conditions when administered in animals in vivo, suggesting their critical pathogenic roles in sepsis [11].

Exercise training is considered to be associated with inflammatory response. Regular exercise attenuates vital organ dysfunction and damages inflicted by LPS-induced sepsis [12]. We have recently reported that extracellular superoxide dismutase, which is increased by exercise training, protects against endotoxemia-induced multiple organ dysfunction syndrome (MODS) in mice [13]. However, the impact of exercise on the prenatal regulation of inflammatory response, particularly in LPS-induced sepsis, remains unclear.

Here, we used voluntary wheel running during pregnancy to test in mice the hypothesis that maternal exercise training reduces inflammatory responses to endotoxin in adult offspring.

## 2 Materials and methods

### 2.1 Animals

**Fig. 1:**
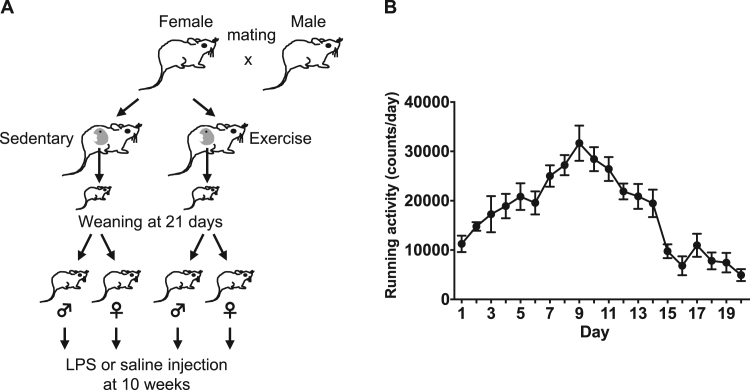
Study design and running activity during pregnancy. A) Study design; B) Running activity during pregnancy.

Eight-week male (*n* = 3) and female (*n* = 13) C57BL/6 J mice were purchased from Japan SLC (Shizuoka, Japan). At the time of mating, a sedentary male mouse was placed in sedentary female cages overnight, and pregnancy was confirmed by a vaginal plug. Mice with plug were assigned into the following two groups: sedentary (Sed; *n* = 6) and exercise training (Ex; *n* = 7). Ex mice were individually housed in cages equipped with running wheels until delivery. The running activity was monitored using a wireless running wheel (Med Associates, Inc., Fairfax, VT) continually during exercise training periods. The running wheels were removed from cages within 12 h after delivery. All dams and offspring were fed normal chow with sedentary activity during lactation and after weaning (at 21 days). At 10 weeks of age, the offspring were assigned into the following four groups: Sed-saline, Sed-LPS, Ex-saline, and Ex-LPS. The offspring in the Sed-LPS and Ex-LPS groups were intraperitoneally (i.p.) injected a lethal (30 mg/kg; male: Sed = 7, Ex = 8; female: Sed = 9, Ex = 7) or nonlethal (15 mg/kg; male: Sed = 7, Ex = 7; female: Sed = 8, Ex = 9) dose of LPS (Sigma-Aldrich, St. Louis, MO) (Fig. 1A). The offspring in the Sed-saline and Ex-saline groups were i.p. injected with a saline (male: Sed = 6, Ex = 6; female: Sed = 7, Ex = 7). Notably, each group comprised offspring from > 5 different dams. All experimental procedures in this study were performed under the approval of the Ethics Committee of Nagoya City University.

### 2.2 Endotoxin exposure

The Sed-LPS and Ex-LPS mice were i.p. injected with a lethal (30 mg/kg) or nonlethal (15 mg/kg) dose of LPS at 10 weeks. The mice injected a lethal dose of LPS were monitored for 60 h to record survival. The body weights of mice that were injected with a nonlethal dose of LPS were measured at 18 h. The plasma, lung, and liver were harvested from the mice after sacrificing them by cervical dislocation under anesthesia. Both the Sed-saline and Ex-saline groups received an equivalent volume of the vehicle.

### 2.3 Cytokine antibody analysis

To determine the volume of multiple cytokine proteins in the blood, mixed plasma samples from all mice in each group were analyzed using cytokine antibody array. Pooled plasma was diluted and subjected to cytokine profiling using the proteome profiler mouse cytokine array (R&D Systems, Minneapolis, MN) according to the manufacturer's instructions. Briefly, membranes were blocked with a blocking reagent, and then 2 ml of pooled plasma samples from each group were individually added and incubated at 4 °C overnight. Membranes were washed and incubated by streptavidin-HRP at room temperature for 30 min. The membranes were incubated with Chemi Reagent Mix and imaged using an ImageQuant LAS 500 (GE Healthcare, Little Chalfont). The images were quantified using Image J software.

### 2.4 Semiquantitative RT-PCR

To assess the inflammatory cytokine mRNA expression, total RNA was isolated from the lung and liver using TRIzol (Invitrogen, Madison, WI) according to the manufacturer's instructions. Reverse transcription was performed with 2 μg of the total RNA using the SuperScript II First-Strand Synthesis System for RT-PCR (Life Technologies, Carlsbad, CA). Semiquantitative RT-PCR analysis was performed to measure *IL-1β*, *IFN-γ*, *Toll-like receptor-4 (TLR-4)*, and *GAPDH* mRNAs. The following PCR primers were used: *IL-1β*: 5′-TGCCACCTTTTGACAGTGATG-3′ and 5′-GGTATTTTGTCGTTGCTTGGTTCT-3′; *IFN-γ*: 5′-AGGAACTGGCAAAAGGATGGT-3′ and 5′-AACCCCGCAATCACAGTCTT-3′; *TLR-4*: 5′-TCCCTGCATAGAGGTAGTTCCTA-3′ and 5′-CCCTGAAAGGCTTGGTCTTGA-3′; and *GAPDH*: 5′-TGAAGTCGCAGGAGACAACC-3′ and 5′-TGAAGTCGCAGGAGACAACC-3′. The template denaturation was performed at 94 °C for 5 min followed by 30 (*IL-1β*), 32 (*IFN-γ*), 29 (*TLR-4*), and 30 (*GAPDH*) cycles consisting of 30 s at 94 °C, 30 s at 60 °C, and 40 s at 72 °C. The PCR products were separated by electrophoresis on 2% agarose gel, stained with AtlasSight DNA Stain (BioAtlas, Tartu, Estonia). The stained gels were analyzed using ImageQuant LAS 500 and quantified using Image J software. Results were normalized by *GAPDH* mRNA and presented as fold change to the lung or liver in sedentary mice injected with normal saline.

### 2.5 Statistical analysis

Body weight, *IL-1β*, and *IFN-γ* mRNA expression were analyzed using two-way ANOVA followed by Tukey's post-hoc test when applicable. *TLR-4* mRNA expression was analyzed using Mann-Whitney test. Survival was analyzed using the log rank test. P < 0.05 was considered statistically significant, and mean values are described along with SE.

## 3 Results

### 3.1 Running activity in pregnant mice

When pregnant mice were subjected to voluntary running, the daily running distance gradually increased, reached a peak level after 9 days, and then gradually decreased until delivery (Fig. 1B).

### 3.2 Maternal exercise training could not prevent lethality during LPS-induced septic shock

**Fig. 2:**
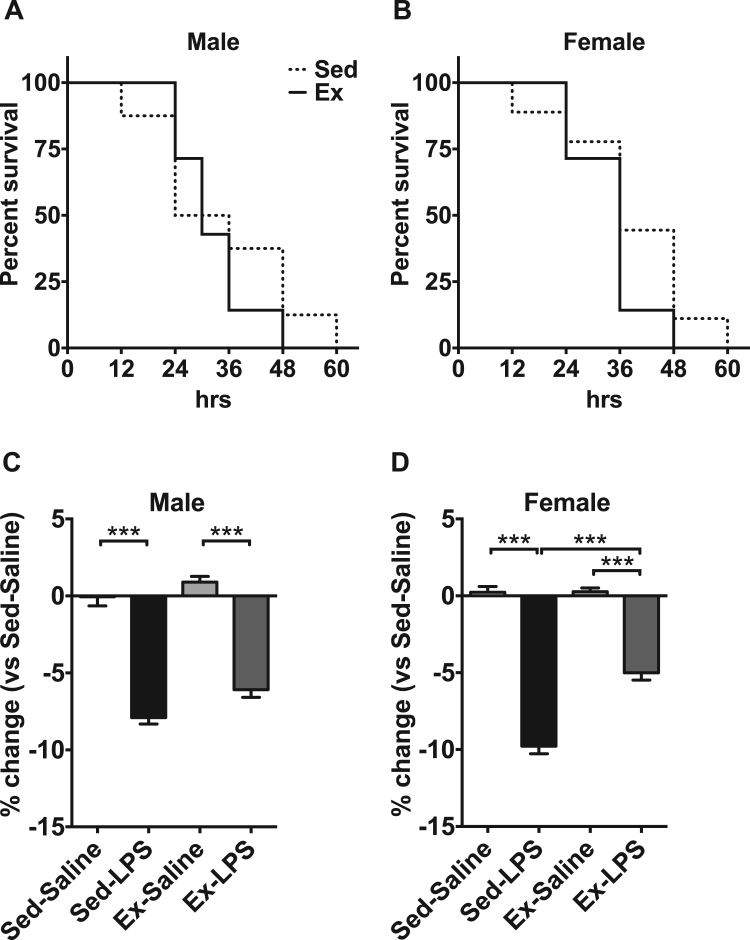
Survival study and change in body weight in a mouse model of LPS-induced sepsis. A) Survival curves for male mice injected (i.p.) with LPS (30 mg/kg); B) Survival curves for female mice injected (i.p.) with LPS (30 mg/kg); C) Change in body weight in male mice injected (i.p.) with LPS (15 mg/kg); D) Change in body weight in female mice injected (i.p.) with LPS (15 mg/kg). ***p < 0.001.

Investigating the role of maternal exercise on the survival in a clinically relevant model of septic shock induced by a lethal dose of LPS revealed that the survival did not significantly differ between offspring from sedentary dams and those from exercise dams (Fig. 2A, B).

### 3.3 Maternal regular exercise is effective against the LPS-induced loss of body weight in female

It is well known that endotoxin exposure generally decreases the body weight. Therefore, we assessed whether maternal exercise could protect offspring from a nonlethal dose of endotoxin-induced loss of body weight. The nonlethal dose of LPS injection significantly decreased the body weight in male and female offspring (Fig. 2C, D). However, female offspring in the Ex-LPS group significantly attenuated the LPS-induced loss of body weight compared with those in the Sed-LPS group (Sed-LPS, − 9.8%; Ex-LPS, − 5.0%) (p < 0.05) (Fig. 2D). No significant differences were observed in male offspring (Fig. 2C).

### 3.4 Maternal regular exercise attenuated the LPS-induced inflammatory cytokines in female offspring

**Fig. 3:**
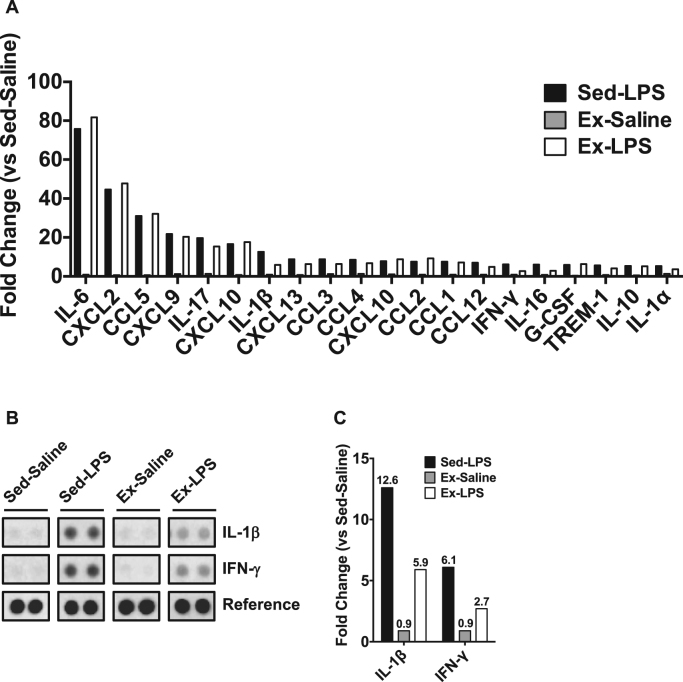
Change in plasma cytokines in a female mice model of LPS-induced sepsis. A) Proteome profiler mouse cytokine arrays were performed to evaluate plasma cytokines; B) Representative images for cytokine antibody array; C) Change in plasma IL-1β and IFN-γ protein in female mice.

Because the LPS-induced loss of body weight is associated with inflammation, we assessed the plasma inflammatory cytokines in female offspring using the cytokine array. The nonlethal dose of LPS injection increased the levels of numerous inflammatory cytokines and chemokines, such as IL-6 (Sed-LPS, 75.8-fold; Ex-LPS, 81.7-fold), MIP-2/CXCL2 (Sed-LPS, 44.6-fold; Ex-LPS, 47.8-fold), and RANTES/CCL5 (Sed-LPS, 31.0-fold; Ex-LPS, 32.1-fold) in the LPS injection group compared with the Sed-saline group (Fig. 3A). Among the inflammatory cytokines elevated by the nonlethal dose of LPS injection in the Sed-LPS group, maternal exercise dramatically attenuated the induction of inflammatory cytokines such as IL-1β (Sed-LPS, 12.6-fold; Ex-LPS, 5.9-fold) and IFN-γ (Sed-LPS, 6.1-fold; Ex-LPS, 2.7-fold) (Fig. 3BC).

### 3.5 Maternal regular exercise attenuated the LPS-induced IL-1β and IFN-γ mRNA expression in lung and liver without inducing TLR4 in female offspring

**Fig. 4:**
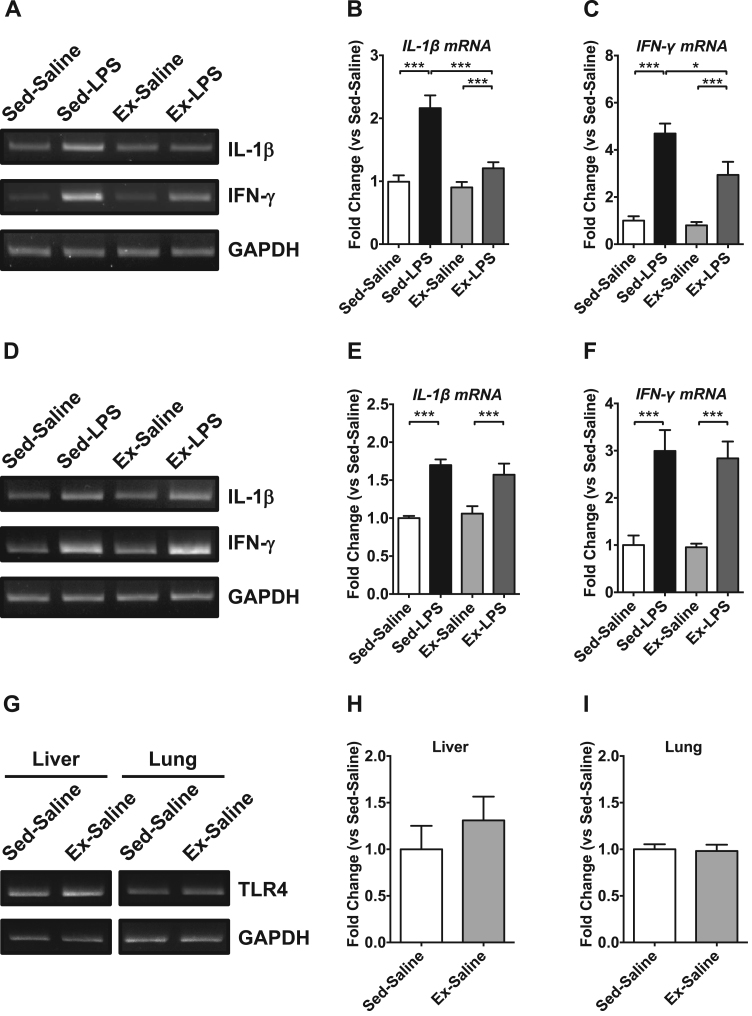
Change in cytokine mRNA expression in the liver and lungs in a female mice model of LPS-induced sepsis. A) Representative image for *IL-1β*, *IFN-γ*, and *GAPDH* mRNA expression in the liver; B) *IL-1β* mRNA expression in the liver; C) *IFN-γ* mRNA expression in the liver; D) Representative image for *IL-1β*, *IFN-γ*, and *GAPDH* mRNA expression in the lung; E) *IL-1β* mRNA expression in the lung; F) *IFN-γ* mRNA expression in the lung; G) Representative image for *TLR4* and *GAPDH* mRNA expression in the liver and lung; H) *TLR4* mRNA expression in the liver; I) *TLR4* mRNA expression in the live. *p < 0.05; *** p < 0.001.

We diagnosed sepsis as previously defined, which often predisposed the failure of vital organs, such as the lung and liver, currently referred to as MODS. Inflammatory cytokines have been reported to play crucial roles in the induction of sepsis and the development of MODS [14]. Therefore, we assessed the *IL-1β* and *IFN-γ* mRNA expression in the lung and liver. The nonlethal dose of LPS injection significantly increased the *IL-1β* and *IFN-γ* mRNA expression in the lung and liver compared with that in the saline injection group (Fig. 4A-F). Among the increased *IL-1β* and *IFN-γ* mRNA expression in the lung and liver by the nonlethal LPS injection, maternal exercise significantly attenuated the induction of *IL-1β* (Sed-LPS, 2.2-fold; Ex-LPS, 1.3-fold) and *IFN-γ* (Sed-LPS, 4.7-fold; Ex-LPS, 3.7-fold) mRNA in the liver (Fig. 4A-C). Although maternal exercise tended to inhibit the induction of lung *IL-1β* and *IFN-γ* mRNA expression, no statistically significant difference was observed between the Sed-LPS and Ex-LPS groups (Fig. 4D-F). Because LPS initiates the innate immune response via the *TLR4* signaling pathway, we assessed *TLR4* mRNA in the lung and liver. Although maternal exercise tended to increase *TLR4* mRNA expression in the liver, no statistically significant difference was observed between the Sed-saline and Ex-saline groups (Fig. 4G-I).

## 4 Discussion

Recent studies have demonstrated that regular exercise during pregnancy prevents several diseases, such as cardiovascular diseases, obesity, and type II diabetes, in adulthood [1], [2]. However, little information is available about the impact of maternal exercise during pregnancy on the regulation of immune responses to infection, particularly sepsis. Our results demonstrated that maternal exercise significantly prevented the LPS-induced loss of body weight and the induction of inflammatory cytokines in female offspring, suggesting that maternal exercise during pregnancy is a potential candidate of the onset of sepsis in offspring.

Inflammatory cytokines have been reported to play crucial roles in the induction of systemic inflammation and development of sepsis [11]. Inflammatory cytokines, such as IL-1β, TNF-α, and IFN-γ, are detected in the blood of patients with sepsis and induce septic shock-like conditions when animals are administrated with these inflammatory cytokines, suggesting that these cytokines have key pathogenic roles in sepsis [15], [16]. In this study, despite the limitation of not assessing plasma levels of individual cytokines by ELISA. We found that a nonlethal dose of LPS, corresponding to an established animal model of sepsis, enhanced the circulating levels of IL-1β and IFN-γ assessed by cytokine array. The induction of IL-1β and IFN-γ by the LPS injection was attenuated in offspring from exercise dams compared with that in offspring from sedentary dams. Although circulating IL-1β and IFN-γ are not the only factors that induce sepsis, our findings suggest that regular exercise during pregnancy is a potential predictor of the onset of systemic inflammation and development of sepsis in offspring.

MODS is defined as a clinical syndrome that is characterized by the progression of potentially reversible physiological dysfunction in more than two organs and is induced by trauma, hemorrhagic shock, and sepsis. Despite being intensively investigated in recent years, MODS remains the primary cause of death in intensive care units with an extremely high mortality rate (30–80%) [17]. The lack of an effective therapy for MODS to date could be attributed to an incomplete understanding regarding the prevention against inflammatory cytokines in sepsis. Therefore, we assessed the expression of inflammatory cytokines mRNA in the lungs and liver and observed that maternal exercise during pregnancy inhibited the expression of LPS-induced inflammatory cytokines mRNA in the liver and lungs. Our findings suggested that regular exercise during pregnancy contributes to the inhibition of severe systemic inflammation. Although the application of our findings to the prevention and treatment of sepsis and MODS is challenging, these could contribute to predicting the onset of sepsis and MODS.

LPS initiates the innate immune response via the TLR4 signaling pathway [18], [19]. Mice with either a defective or a disrupted TLR4 fail to respond to LPS and are more susceptible to bacteremia [20], [21]. In contrast, the enhanced expression of TLR4 enhances the sensitivity to LPS [22]. In this study, we measured the TLR4 mRNA expression in the lung and liver and found that this expression was not significantly different between the Sed-LPS and Ex-LPS groups, which suggested that the suppressed inflammatory response observed in the Ex-LPS group is not limited to the LPS-TLR signaling pathway.

A promising factor for the inhibition of inflammatory response in offspring by maternal exercise training is the epigenetic modification of essential inflammatory genes through DNA methylation [23]. DNA methylation typically occurs in differentiated cells at the cytosine of CpG dinucleotide pairs. The methylation of CpG islands can impair transcription factor binding and can stably silence gene expression [24]. Recently, we reported that regular exercise during pregnancy prevents the maternal high-fat diet–induced hypermethylation of the pgc-1α gene and the age-dependent metabolic dysfunction in offspring [6]. Epigenetic modification by promoter methylation, which regulates inflammatory cytokines, such as NF-kB, could be an important consequence relevant to the development of sepsis.

Another potential mechanism for the reduced inflammatory response in offspring from exercising dams is endotoxin tolerance. Previous studies reported that exercise induces increases in LPS absorption from the gastrointestine to the circulation [25]. Long-term exposure to LPS causes immune cells to enter an immunosuppressive state, which make them less responsive to subsequent exposures to LPS. Such phenomenon is referred to as endotoxin tolerance [26]. Therefore, it is plausible that maternal exercise induced low-dose endotoxemia during pregnancy, leading to an endotoxin tolerant state in the offspring.

The maternal exercise-induced protection against septic shock-induced loss of body weight was not as evident in males in comparison to females. In addition, a potential inhibition of inflammatory cytokines following LPS exposure in the male offspring of exercising dams was not examined. Recent studies have revealed that early environmental exposure affects sex-dependent effects on the immune function. These sex-based immunological differences contribute to variations, such as autoimmune diseases, malignancies, and the susceptibility to infectious diseases [27]. Nevertheless, further investigations, such as the exercise-induced modification of environment during pregnancy, are warranted to elucidate the sex distinction in response to LPS.

Among the inflammatory cytokines elevated by the nonlethal dose of LPS in the Sed-LPS group, maternal exercise also attenuated the induction of CCL3 (Sed-LPS, 8.8-fold; Ex-LPS, 6.4-fold), IL-16 (Sed-LPS, 6.0-fold; Ex-LPS, 3.0-fold), and IL-1α (Sed-LPS, 5.3-fold; Ex-LPS, 3.6-fold)in their offspring. However, future detailed analyses of CCL3, IL-16, and IL-1α in this setting might provide additional insights into potential mechanisms mediating the observed protection against endotoxemia.

We cannot dismiss the possibility that maternal exercise also inhibited the LPS-induced induction of IL-1β and IFN-γ concentration in male offspring from Ex mice. Cytokine analysis in male mice should provide further evidence for our conclusion.

In conclusion, this study revealed that maternal exercise inhibits the LPS-induced inflammatory response in female offspring, thereby suggesting that regular exercise during pregnancy is a determining factor for the predisposition of offspring to sepsis.
