# Supplementary material for: Maternal exercise training attenuates endotoxin-induced sepsis in mice offspring
Source: Biochem Biophys Rep. 2018 Jun 14;15:19–24. doi: 10.1016/j.bbrep.2018.06.001 (PMC6008276; doi:10.1016/j.bbrep.2018.06.001)
Supplement: Supplementary file 1 — Supplementary material [file mmc1.docx]

**Conflicts of interest**

We have no conflict of interest.
